# Neutrophil-mediated experimental metastasis is enhanced by VEGFR inhibition in a zebrafish xenograft model

**DOI:** 10.1002/path.4013

**Published:** 2012-07-03

**Authors:** Shuning He, Gerda EM Lamers, Jan-Willem M Beenakker, Chao Cui, Veerander PS Ghotra, Erik HJ Danen, Annemarie H Meijer, Herman P Spaink, B Ewa Snaar-Jagalska

**Affiliations:** 1 Institute of Biology, Leiden UniversityEinsteinweg 55, 2333 CC Leiden, The Netherlands; 2 Leiden Institute of Physics, Leiden University2300 RA Leiden, The Netherlands; 3 Division of Toxicology, Leiden/Amsterdam Centre for Drug Research, Leiden University2300 RA, The Netherlands; 4 Department of Pediatric Oncology, Dana-Farber Cancer Institute, and Children's Hospital Boston, Harvard Medical SchoolBoston, MA 02115, USA

**Keywords:** zebrafish, neutrophils, macrophages, tumour vascularization, micrometastasis, VEGFR inhibition

## Abstract

Inhibition of VEGF signalling effectively suppresses localized tumour growth but accelerates tumour invasiveness and micrometastasis by unknown mechanisms. To study the dynamic and reciprocal interactions between tumour cells and their microenvironment during these processes, we established a xenograft model by injecting tumour cells into the blood circulation of transparent zebrafish embryos. This reproducibly results in rapid simultaneous formation of a localized tumour and experimental micrometastasis, allowing time-resolved imaging of both processes at single-cell resolution within 1 week. The tumour vasculature was initiated *de novo* by remodelling of primitive endothelial cells into a functional network. Roles of myeloid cells in critical tumourigenesis steps such as vascularization and invasion were revealed by genetic and pharmaceutical approaches. We discovered that the physiological migration of neutrophils controlled tumour invasion by conditioning the collagen matrix and forming the metastatic niche, as detected by two-photon confocal microscopy and second harmonic generation. Administration of VEGFR inhibitors blocked tumour vascularization and a localized tumour growth but enhanced migration of neutrophils, which in turn promoted tumour invasion and formation of micrometastasis. This demonstrates the *in vivo* cooperation between VEGF signalling and myeloid cells in metastasis and provides a new mechanism underlying the recent findings that VEGFR targeting can promote tumour invasiveness. Copyright © 2012 Pathological Society of Great Britain and Ireland. Published by John Wiley & Sons, Ltd.

## Introduction

Vascular endothelial growth factor (VEGF) is an essential mediator of vascularization under both physiological and tumour conditions. Due to the control function of tumour vascularization in development of both primary and secondary tumours 1, VEGF signalling is one of the leading therapeutic targets for cancer treatment 2. Unfortunately, clinical results of anti-angiogenic therapies targeting the VEGF receptor tyrosine kinase (VEGFR) or VEGF have been more modest than predicted by initial preclinical studies, and frequently effects on overall patient survival have been absent or minor 2–6. Recent preclinical and clinical studies have shown that, despite the suppression of localized primary tumour growth, inhibition of VEGF signalling can promote tumour invasiveness and elicit early-stage micrometastasis 2, 7–12. Although the underlying mechanism is not yet clear, the tumour microenvironment, as the primary target of the anti-angiogenic agents, is thought to play an important role in the elevated malignancy 3, 13, 14.

Tumour progression is controlled by dynamic and reciprocal interactions between malignant tumour cells and non-malignant cells in their microenvironment 13, 14. Tumour cells secrete pro-angiogenic factors, including VEGF, to activate the endothelial cells of neighbouring blood vessels and trigger angiogenesis to form tumour neovasculatures 15. The tumour stromal cells, including fibroblasts and bone marrow-derived cells (BMDCs), can secrete pro-angiogenic factors and control the angiogenic switch 16, 17. The myeloid lineages of BMDCs can be recruited by tumour cells to enhance their survival and aggressiveness 15. It has been shown that CD11b^+^ Gr1^+^ myeloid cells, which include neutrophils, macrophages and myeloid-derived suppressor cells, promote tumour progression independent of the adaptive immune system, and that these cells render tumours refractory to angiogenic blockade by VEGF antibodies 18. They have also been implicated in the formation of the premetastatic niche 19. However, as these cells are usually located in deep tissues and can be functionally affected by invasive detection methods, *in vivo* kinetic studies of their actual roles in tumour progression remain challenging. Therefore, non-invasive visualization of the kinetic interaction between tumour cells and their microenvironment at high resolution will largely improve our understanding of basic cancer biology and will help to design new therapeutic strategies.

The zebrafish, *Danio rerio*, has been an important animal model for cancer, immune and stem cell research over the last decade 20–23. Many molecular and cellular components that operate during tumourigenesis are conserved between zebrafish and mammals. A wide range of pharmacologically active compounds elicit physiological responses in zebrafish embryos comparable to those in mammalian systems 24, making zebrafish ideal for identifying clinically relevant genes and compounds that regulate tumour progression. The transparency of zebrafish, in combination with the availability of various tissue-specific fluorescent reporter transgenic lines 25, 26, allows high resolution *in vivo* analysis of tumour progression and the interactions between tumour cells and the host microenvironment 27, 28. Several tumour transplantation assays with human and mammalian cells have been developed to study different aspects of tumour malignancies in embryo and adult zebrafish, such as tumour cell migration, proliferation, angiogenesis and tumour cell extravasation 25, 27–31. However, most of these assays are limited to one selected step of tumour development and do not represent the full complexity of tumourigenesis in one model. In addition, for zebrafish embryonic engraftment models there are no reports published describing tumour cells extravasation from the circulation and invasion into the surrounding tissue where cells proliferate to form experimental metastases.

We have established a rapid, reproducible zebrafish embryonic xenograft model for simultaneous formation of a localized tumour and experimental micrometastasis by intravascular injection of tumour cells into the blood circulation of zebrafish embryos. With non-invasive high-resolution imaging we characterized the critical steps of tumour progression, including tumour vascularization and tissue invasion. Using this model, we found that myeloid cells are involved in these tumour processes, and especially that neutrophils condition the collagen matrix to facilitate metastatic niche formation and tumour invasion. Importantly, we show that VEGFR inhibitors suppress localized tumour growth but, in contrast, promote tumour invasion and micrometastasis formation by enhancing neutrophil migration.

## Materials and methods

### Zebrafish maintenance, morpholino injection and pharmacological treatment

Zebrafish and embryos were raised, staged and maintained according to standard procedures in compliance with the local animal welfare regulations. The transgenic lines Tg(fli1:GFP) and Tg(mpx:GFP)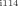 were used in this study 25, 26. 0.2 mm *N*-phenylthiourea (PTU; Sigma) was applied to prevent pigment formation from 1 day post-fertilization (dpf). For Pu.1 knockdown as previously published, Pu.1 MO (1 mm for partial knockdown and 2 mm for complete knockdown 32 was injected into the yolk at the one-cell stage, as described 33. For pharmacological inhibition, the VEGFR tyrosine kinase inhibitors KRN633 (0.1–1.0 µm; Santa Cruz) or Sunitinib (0.1–1.0 µm; Sigma), beclomethasone (25 µm; Sigma) and β-amino- proprionitrile (βAPN, 500 µm; Sigma) were applied directly to the egg water and refreshed every 2 days.

### Cell lines

Murine aortic endothelial (MAE) cells and tumourigenic FGF2-over-expressing FGF2-T-MAE cells 34 (provided by M. Presta, University of Brescia, Italy) and the human breast carcinoma cell line MDA-MB-231 35 (provided by P. ten Dijke, Leiden University Medical Centre, The Netherlands) were cultured as previously described. 4T1 (CRL-2539, ATCC) and PC3 (CRL-1435, ATCC) cells were cultured in RPMI 1640 supplemented with 10% fetal bovine serum (FBS; Invitrogen). Stable fluorescent cell lines were generated using lentiviral vectors expressing dsRed or mCherry (provided by R. C. Hoeben, Leiden University Medical Centre, The Netherlands). The virus-infected cells were used for experiments without FACS sorting and the fluorescence was stable *in vitro* for > 10 passages. Zebrafish fibroblast cell lines ZF4 and PAC2 were cultured as previously described 36.

### Embryo preparation and tumour cell implantation

Dechorionized 2dpf zebrafish embryos were anaesthetized with 0.003% tricaine (Sigma) and positioned on a 10 cm Petri dish coated with 1% agarose. Mammalian cells were trypsinized into single cell suspensions, resuspended in phosphate-buffered saline (PBS; Invitrogen), kept at room temperature before implantation and implanted within 3 h. Non-fluorescent cells were labelled with the fluorescent cell tracker CM-DiI (Invitrogen) according to the manufacturer's instructions. The cell suspension was loaded into borosilicate glass capillary needles (1 mm o.d. × 0.78 mm i.d.; Harvard Apparatus) and the injections were performed using a Pneumatic Pico pump and a manipulator (WPI). 50–400 cells, manually counted, were injected at approximately 60 µm above the ventral end of the duct of Cuvier where it opens into the heart. After implantation with mammalian cells, zebrafish embryos (including non-implanted controls) were maintained at 34 °C to compromise between the optimal temperature requirements for fish and mammalian cells 37. Up to 600 implantations were manually achieved per h, with survival rates of > 90% until 6 days post-implantation (dpi). For pharmacological inhibition, beclomethasone was applied to the embryos 4 h before implantation and KRN633, Sunitinib and βAPN were applied 4–6 h post-implantation. For each cell line or condition, data are representative of ≥ three independent experiments, with ≥ 30 embryos/group. Experiments were discarded when the survival rate of the control group was < 90%.

### Microscopy and analysis

For live imaging, embryos were anaesthetized using 0.016% tricaine (Sigma) and mounted in 0.6% low-melting agarose. Fixed embryos were imaged in PBST. Fluorescent image acquisition was performed using a Leica MZ16FA stereo microscope, a Leica TCS SPE confocal microscope or a Zeiss LSM exciter on an Axio Observer confocal microscope. Confocal stacks were processed for maximum intensity projections with Zeiss ZEN2009 software or ImageJ software. Images were adjusted for brightness and contrast using ImageJ. Overlays were created using Adobe Photoshop CS4 or ImageJ. 3D reconstructions and movies were assembled using ImageJ.

### Second-harmonic generation and two-photon microscopy

Second-harmonic generation (SHG) was used to image the helical structures of collagen fibres, which are capable of combining two photons into one 38. The two-photon microscopy was performed on a Zeiss 710 NLO upright confocal microscope equipped with a Spectraphysiscs Deep See MP laser. The images were obtained with an excitation wavelength of 750 nm and only emitted light with a wavelength in the range 371–467 nm was detected.

### Myeloid cell detection

In the Tg(fli1:GFP) line, the neutrophils in fixed embryos were detected using the Peroxidase/ Myeloperoxidase Leukocyte Kit (Sigma), as described previously 39. The embryos were imaged with transmitted light and the black-stained cells were extracted using the Threshold function of ImageJ and subsequently converted into a selected colour for overlay with fluorescence images. For macrophage detection, immunohistochemistry for L-plastin was performed after the myeloperoxidase activity assay and the Mpx^−^/L-plastin^+^ cells were counted as macrophages 31. The Tg(mpx:GFP)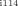 line was used to monitor neutrophil migration *in vivo* 26. Time-lapse imaging (1 min intervals, ≥ 30 min) was performed using a Leica stereofluorescence microscope. The migration tracks were generated by maximum intensity projections of the time stacks, using ImageJ. For individual neutrophil tracking, the first 20 GFP^+^ cells from the posterior end of the tail fin in each embryo, which exhibited consistent intensity for at least 15 min, were tracked and analysed using the Manual Tracking plugin of ImageJ.

### Immunohistochemistry

Whole-mount immunohistochemistry was carried out as described 40. Primary antibodies and dilutions were used as follows: L-plastin (rabbit anti-zebrafish, 1:500; provided by A Huttenlocher 31), ZO-1 (mouse anti-human, 1:300; Invitrogen), phosphohistone H3 (rabbit anti-human, 1:500; Santa Cruz). A 1:200 dilution of the secondary antibodies (Alexa 405 anti-mouse, Alexa 568 anti-mouse or Alexa 568 anti-rabbit; Invitrogen) was used for detection.

### Tail fin wounding

Zebrafish embryos at 2–3 dpf were anaesthetized using 0.016% tricaine (Sigma) and then wounded on the ventral side of the tail fin with the tip of a glass capillary needle, as described 39. Wounded embryos were fixed in 4% paraformaldehyde/PBS at various time points after the wounding and stored in PBS containing 0.01% Tween-20 (PBST) at 4 °C for myeloid cell detection.

### Microangiography

Tetramethylrhodamine dextran was injected into the sinus venosus of anaesthetized zebrafish embryos, as described 41. Images were acquired within 10 min after injection.

### Statistical analysis

Statistical analysis was performed using Prism 4 software (GraphPad), two- or one-tailed unpaired *t*-tests with confidence intervals of 90% or 95%: ns, not significant (*p* > 0.05); *0.01 < *p* < 0.05; **0.0001 < *p* < 0.01; ***p < 0.0001.

## Results

### Intravascular injection of tumour cells into zebrafish embryos resulted in localized tumour growth and micrometastasis within 6 days

**Figure 1:**
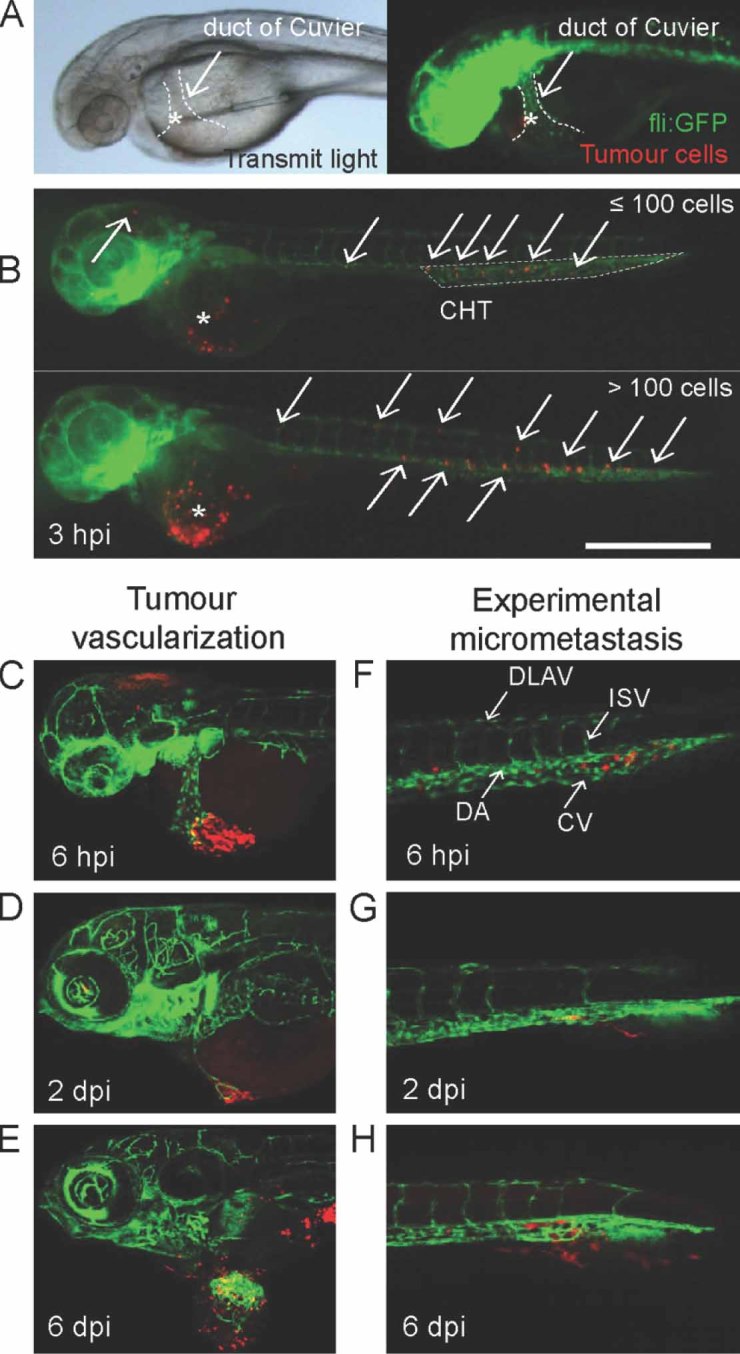
Intravascular injection of tumour cells into zebrafish embryos resulted in localized tumour growth and micrometastasis within 6 days. (A) Bright-field (left) and fluorescence (right) images showing the injection of tumour cells (red) into the zebrafish embryonic circulation (green) via the ventral side of the duct of Cuvier. (B) Dissemination of implanted tumour cells in embryos injected with ≤100 (top) or > 100 cells. Dissemination was observed in almost all embryos (124/125) at 3hpi. *Injection sites; arrows, disseminated tumour cells; the caudal hametopoietic tissue (CHT) is outlined (top). Note that tumour cells also accumulated close to the injection site in the example of an embryo injected with > 100 cells (bottom). Scale bar = 1 mm. (C–E) Progression of tumour formation. Tumour cells accumulated close to the injection site at 6hpi (C), induced formation of neovasculature at 2dpi (D) and displayed localized tumour growth at 6dpi (E). (F–H) Formation of micrometastases. Disseminated tumour cells resided in the tail at 6hpi (F), invaded into the neighbouring tail fin at 2dpi (G) and formed micrometastasis colonies in the fin tissue at 6dpi (H). Images in (A, B) were acquired using a Leica MZ16FA stereo microscope; those in (C–H) were acquired using a Leica TCS SPE confocal microscope with a × 20 dry objective. Red in (A, B, E), tumour cells labelled with the red cell tracker CM-DiI; red in (C, D, F–H), tumour cells stably expressing dsRed; green in all, GFP-endothelial cells of the Tg(Fli:GFP) line. DLAV, dorsal longitudinal anastomotic vessel; ISV, intersegmental vessel; DA, dorsal aorta; CV, caudal vein. Data are representative images of > 20 independent, highly reproducible experiments

In order to develop an experimental metastasis model, as frequently used in mice, we implanted up to 400 fluorescent tumour cells into the blood circulation of zebrafish embryos at 2 dpf. The tumour cells haematogenously disseminated in the embryo immediately after injection into the duct of Cuvier; a wide circulation channel on the embryonic yolk sac connecting the heart to the trunk vasculature (Figure 1A, B). When > 100 tumour cells were injected, the embryonic circulation was not sufficient to disseminate the entire bulk of implanted cells, resulting in the accumulation of non-disseminated cells close to the injection site at the duct of Cuvier (Figure 1B). Using a Tg(Fli:GFP) endothelial reporter transgenic zebrafish line with fluorescent vasculature 25, we found that the accumulated tumour cells induced neovasculature formation and developed into a localized tumour (Figure 1C–E), whereas the disseminated tumour cells formed micrometastic colonies in the trunk of the fish (Figure 1F–H). Localized tumour growth and experimental micrometastases were also induced by several other types of mouse- or human-originated tumourigenic cell lines, but never by non-malignant cell lines (Table 1). This novel experimental metastasis assay bypasses the primary tumour stage and intravasation, but opens the possibility of using this model to screen for antimetastatic agents in a few days, instead of weeks in rodent models.

**Table 1:** Intravascular injection of different cell lines into 2dpf zebrafish embryos

Cell line	Description	Vascularization/localized tumour growth (%)	Tumour invasion/ micrometastasis (%)	*n*
FGF-T-MAE	MAE cells transformed by FGF2 over-expression	97.0	32.8	67
4T1	Mouse breast cancer	64.7	15.5	142
MDA-MB-231	Human breast cancer	0	47.9	48
PC3	Human prostate cancer	25.0	0	60
MAE	Mouse endothelial	0	0	> 100
ZF4/PAC2	Zebrafish fibroblast cell lines	0	0	> 100

For each cell line, data are representative of ≥ three independent experiments (each *n* > 45). The migratory potential for all tumour cells used in Table 1 was detected at 6dpi after yolk implantation of 2dpf embryos (data not shown 37).

### Tumour vascularization is the critical step for localized tumour growth

**Figure 2:**
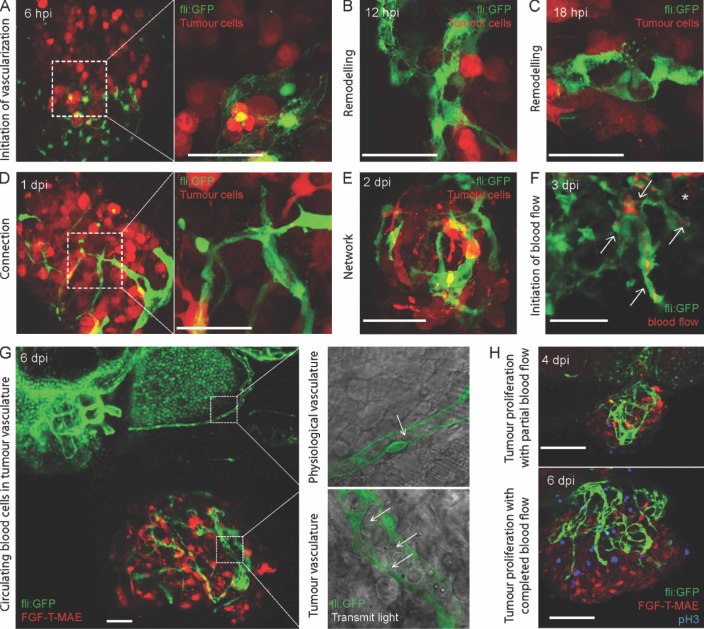
Tumour vascularization and localized tumour growth. (A–E) Initiation of tumour-induced neovasculature. Fli: GFP embryos were imaged from the ventral side to achieve a top view of dsRed-labelled FGF-T-MAE tumour cells, which, after implantation, accumulated under the primitive endothelial cells of the duct of Cuvier (A; 6hpi, with magnification in the right panel). At 12hpi, the primitive endothelial cells in close association with the tumour cells started to change morphology (B). At 18hpi, the primitive endothelial cells were remodelled into vessel-like structures (C; three connected GFP-endothelial cells). The remodelled endothelial cells were further connected within the tumour cell mass at 1dpi (D, with magnification in the right panel). At 2dpi, a partially lumenized vasculature network had formed within the tumour cell mass (E). (F) Microangiography with tetramethylrhodamine dextran showing partial blood flow in the tumour vessels at 3dpi (red, indicated by arrows). *Some leakage of the tumour vessels was also observed. (G) An expanded tumour mass of FGF-T-MAE-mCherry cells, supported by a functional vasculature network, at 6 dpi. Right panel shows magnifications of a pectoral fin vessel (top) and a tumour vessel (bottom). Circulating blood cells within the vessels are indicated by arrows (overlay of transmitted light and GFP channel). (H) Phosphohistone H3 (pH3, blue) staining of Fli:GFP embryos implanted with FGF-T-MAE-mCherry cells. The pH3^+^ staining, as a marker of proliferation, was increased in both mCherry^+^ and mCherry^−^ cell populations after 4dpi, when circulating blood cells started to be detected in the tumour neovasculature. Data are representative images of > 10 independent, reproducible experiments (each *n* > 30). Images in (A–E, H) were acquired using a Zeiss LSM exciter on an Axio Observer confocal microscope with a × 20 dry objective or a × 63 water objective; those in (F, G) were acquired using a Leica TCS SPE confocal microscope with a × 63 water objective. Scale bars = 50 µm

Taking advantage of the transparency of zebrafish embryos, we performed high-resolution confocal imaging to characterize the localized tumour growth. Shortly after implantation, the non-disseminated tumour cells accumulated locally in close association with the primitive endothelial cells of the duct of Cuvier (Figure 2A), which were subsequently remodelled into neovessel-like structures by the tumour cells (Figure 2B, C). Subsequently the neovessels connected and gradually formed an irregular, tortuous, lumenized vasculature network within 2 days (Figure 2D, E). Partial blood flow was detected in the tumour vasculature by microangiography from 3dpi (Figure 2F) and circulating erythrocytes were observed in the lumen after 4dpi (Figure 2G).

Notably, only in the embryos where such functional tumour neovasculature had formed did the localized tumour cells proliferate and form a tumour mass of > 0.15 mm diameter by 6dpi. Phosphorylation of histone H3 (pH3), a proliferation marker, was significantly increased in both fluorescence-positive tumour cells and in fluorescence-negative stromal cells of the localized tumour mass after 4dpi (Figure 2H), when the tumour vasculature started to be functional. These results indicate that successful tumour vascularization was the critical step for localized tumour growth in our model. Importantly, tumour-induced vascularization in our model is the first example of *de novo* vascularization in a zebrafish xenograft system. This phenomenon is fundamentally different from tumour vascularization in a previously described angiogenesis assay, which monitors growth of new vessels from existing vessels towards the tumour implant 34.

### Tumour cell invasion is the critical step for micrometastasis formation

**Figure 3:**
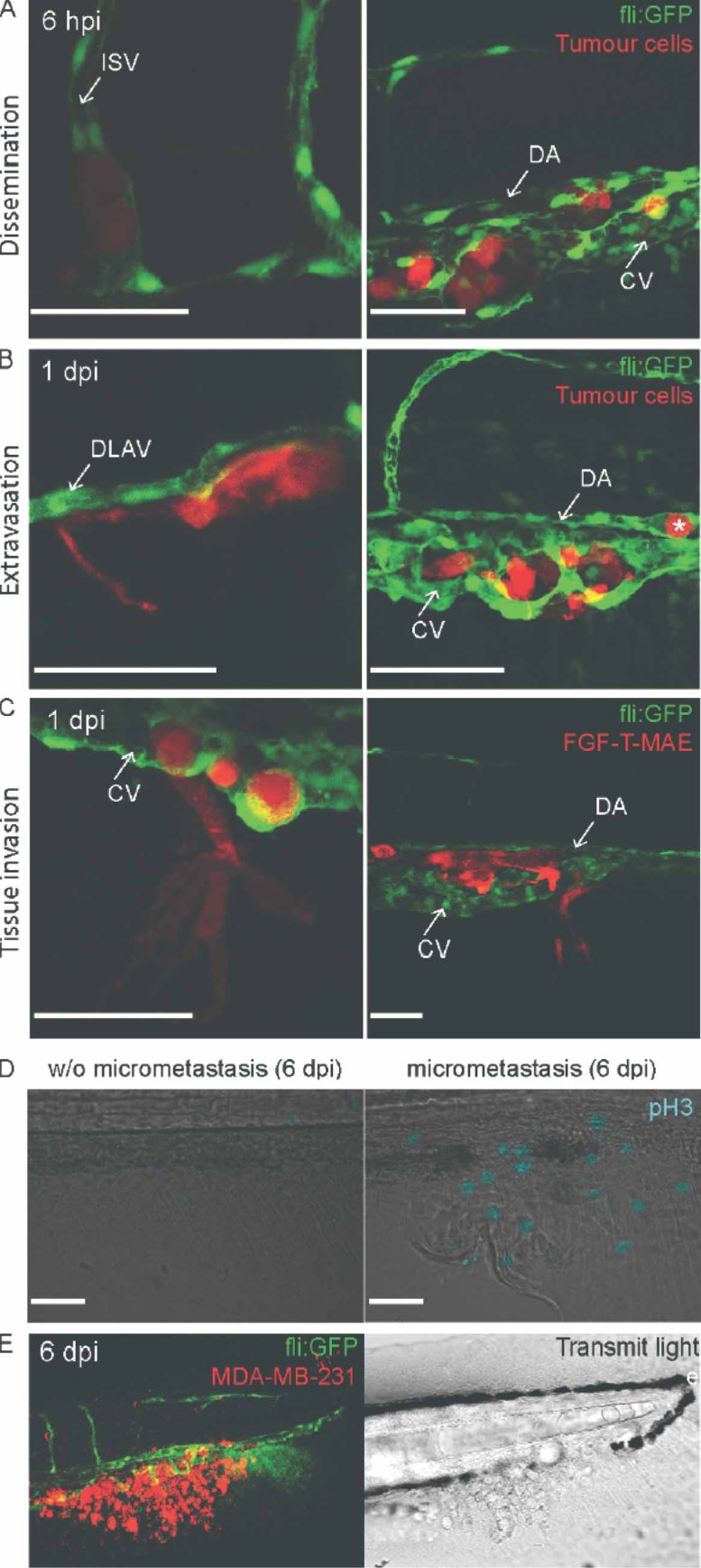
Tumour cell extravasation, invasion and formation of micrometastases. (A) DsRed-labelled FGF-T-MAE cells detected at 6 hpi within the intersegmental vessel (ISV, left), the dorsal aorta (DA, right) and the caudal vein (CV, right). (B) Multiple dsRed-labelled FGF-T-MAE cells having extravasated from the DLAV (left), dorsal aorta (right) and caudal vein (right) at 1 dpi. Only one cell remained in the dorsal aorta (indicated by *). (C) Two representative examples of FGF-T-MAE tumour cell invasion from the posterior end of the caudal haematopoietic tissue into the tail fin at 1 dpi. (D) A cluster of > 35 MDA-MB-231 cells (red, CM-DiI labelled) resident in the tail fin at 6dpi (left, fluorescence; right, transmitted light). (E) Phosphohistone-H3-positive (shown in blue) proliferation of tumour cells after micrometastasis formation: (left) tail fin without micrometastasis; (right) tail fin with micrometastasis. Data are representative images of > 10 independent, reproducible experiments (each *n* > 30) all using Tg(fli–GFP) zebrafish embryos. Images in (A–C) were acquired using a Zeiss LSM exciter on an Axio Observer confocal microscope with a × 63 water objective; (D, E) were acquired using a Leica TCS SPE confocal microscope with a × 20 dry objective. Scale bars = 50 µm

The occurrence of micrometastasis, independent of the localized tumour growth, was also characterized by high-resolution confocal imaging. Micrometastases originated from tumour cells disseminated haematogenously directly after implantation. Disseminated cells were arrested in the host vasculature in the first hour (Figure 3A), and extravasation was detected from 12hpi (Figure 3B). Perivascular tumour cells were observed in multiple foci, including the optic veins, the inter-segmental vessels, the dorsal aorta and the caudal vein (Figure 3B). Most of the perivascular fluorescent signals disappeared before 4dpi, indicating the regression of extravasated cells without initiating tissue invasion and proliferation. However, exclusively at the posterior ventral end of the caudal haematopoietic tissue (CHT, as indicated in Figure 1B) in the tail, perivascular tumour cells were able to invade into the neighbouring tail fin within 1dpi (Figure 3C).

The spatially specific invasion site was validated by other tested tumour cell lines (Figures 1F, H, 3E, Table 1). Such tumour cell invasion was the critical step for micrometastasis formation, as pH3-positive proliferating cells were only detected after successful invasion and formation of 3–50 cell micrometastases in the tail fin (Figure 3D). No angiogenesis towards the micrometastases was observed before the termination of experiments at 6dpi. Experiments were terminated at this stage to comply with European animal regulations. When the embryos were maintained under hypoxic conditions after implantation, there was no significant increase of invasion compared with normoxic conditions (unpublished data). Taken together, dissemination and extravasation events were not site-specific, while tumour cell invasion and formation of micrometastic colonies occurred only at posterior end of the caudal haematopoietic tissue (equivalent to human bone marrow).

### Myeloid cells are involved in tumour vascularization and invasion

**Figure 4:**
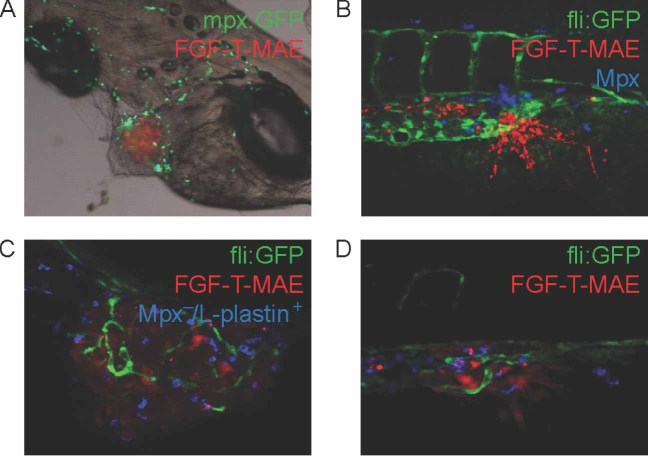
Myeloid cells associated with tumour cells at the localized tumour and micrometastasis sites. (A) Neutrophils [green; Tg(mpx:GFP)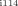] associated with FGF-T-MAE tumour cells (red) at the localized tumour growth site. (B) Neutrophils (blue; Mpx^+^ histochemical staining) associated with the tumour cells (red) at the micrometastasis site. (C, D) Macrophages (blue; Mpx^−^/L-plastin^+^) associated with the tumour cells (red) at the localized tumour (C) and micrometastasis (D) sites. Data are representative images of > 10 independent, reproducible experiments (each *n* > 30). Image in (A) was acquired using a Leica MZ16FA stereo microscope (bright-field/fluorescence overlay image); those in (B–D) were acquired using a Leica TCS SPE confocal microscope with a × 20 dry objective

Leukocytes are known to contribute to different steps of tumour progression, including the formation of tumour vasculature and metastasis 14, 16–18. In zebrafish embryos, the caudal haematopoietic tissue (CHT) is known as a transient site of haematopoiesis and leukocyte differentiation 29. Our observation that tumour cell invasion occurred at the posterior end of the CHT suggested the involvement of leukocytes in tumour progression. In the first few days of zebrafish development, macrophages and neutrophils are the only populations of functional leukocytes in the embryos 30. Using a combination of myeloid lineage markers (Mpx^+^ for neutrophils and Mpx^−^/L-plastin^+^ for macrophages 31), we detected that FGF-T-MAE tumour cells were accompanied by cells of the myeloid lineages, both at the localized tumour growth site near the site of implantation and at the invasion site at the posterior end of the CHT (Figure 4).

**Figure 5:**
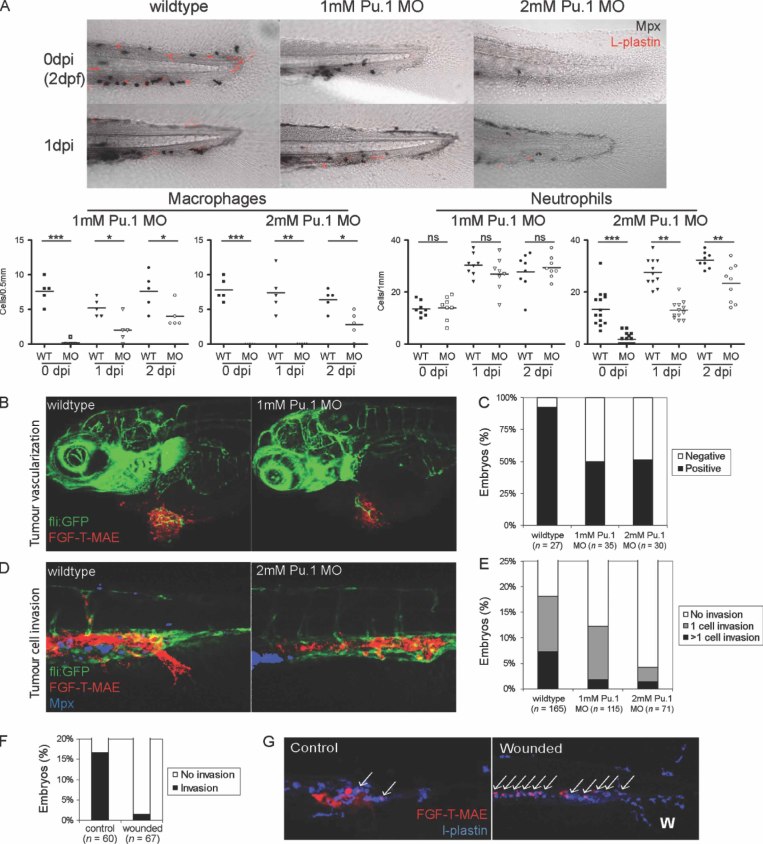
Myeloid cells contribute to tumour vascularization and invasion. (A) Lineage-specific depletion of macrophages and neutrophils by dose-dependent morpholino-mediated knockdown of Pu.1. Neutrophils are shown in black (Mpx^+^ histochemical staining) and macrophages are shown in red (Mpx^−^/L-plastin^+^). In each embryo, the macrophages in the region 0.5 mm from the posterior end of the tail fin and the neutrophils in the region 1 mm from the posterior end of the tail fin were quantified, which are sufficient to represent each linage distribution in the entire embryo. (B, C) Suppression of FGF-T-MAE tumour vascularization under partial (1 mm) or complete (2 mm) knockdown of Pu.1 was significant (Wilcoxon test, *p* < 0.01). Representative embryos with tumour vascularization in the control and Pu.1 knockdown conditions at 3dpi are shown in (B) and quantification of tumour vascularization at 1dpi are shown in (C). Data are representative of > three independent experiments (each *n* ≥ 27). (D, E) Suppression of FGF-T-MAE tumour cell invasion at the posterior end of the CHT under complete knockdown of Pu.1 was significant (Wilcoxon test, *p* < 0.01). Representative control and Pu.1 knockdown embryos at 1dpi (D) and embryos scored for tumour cell invasion at 1dpi (E). Data are representative of > three independent experiments (each *n* > 70). (F) Suppression of tumour cell invasion by wound inflammation in the tail fin at 1dpi was significant (Wilcoxon test, *p* < 0.001). A wound in the tail fin was made mechanically (as in Figure 7D) just after implantation of tumour cells at 2dpf. Data are representative of three independent experiments (each *n* ≥ 60). (G) Phagocytosis of tumour cells (indicated by arrows) around the invasion site by myeloid cells in embryos in which the tail fin was wounded (positioned by ‘W’); tumour cells are shown in red and myeloid cells were stained for L-plastin (blue). Images are representative of > three independent experiments (each *n* > 30). Images in (A, B, D) were acquired using a Leica TCS SPE confocal microscope with a × 20 dry objective; those in (G) were acquired using a Zeiss LSM exciter on an Axio Observer confocal microscope with a × 20 dry objective

To investigate the role of myeloid cells in tumour progression, we used morpholino oligonucleotides to knock down the transcription factor Pu.1, which controls the development and differentiation of myeloid cells in zebrafish embryos 32. As previously shown, Pu.1 knockdown is most effective in suppressing macrophage differentiation, while neutrophil differentiation can be suppressed only under maximal knockdown conditions 42. In agreement, in our experiments macrophages were depleted in zebrafish embryos up to 3dpf by a partial knockdown of Pu.1 (1 mm morpholino; Figure 5A), while neutrophils were depleted by the complete knockdown of Pu.1 (2 mm morpholino; Figure 5A). Using these different doses of Pu.1 morpholino, we found that the formation of tumour neovasculature, but not the physiological vasculature, was impaired under both partial and complete knockdown conditions of Pu.1 (Figure 5B, C), suggesting a major role of macrophages in the tumour vascularization. In contrast, tumour cell invasion at the posterior end of the CHT was only suppressed by complete knockdown of Pu.1, not by partial knockdown (Figure 5D, E), indicating that neutrophils play a predominant role in tumour cell invasion and micrometastasis. Taken together, myeloid cells are involved in the processes of both tumour vascularization and invasion, the critical steps towards localized tumour growth and micrometastasis.

A previous study of zebrafish myeloid cells sensing of HRAS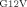-transformed cells suggested homologies between the myeloid cell responses towards transformed cells and wound-induced inflammation 27. Therefore, we investigated the influence of wound-induced inflammation on tumour progression in our model. When the embryos were wounded at the tail-fin, we observed that tumour cell invasion was largely suppressed or abolished (Figure 5F). This suppression of tumour invasion was probably caused by inflammation-enhanced myeloid cell phagocytosis, as the majority of implanted tumour cells were phagocytosed by L-plastin^+^ myeloid cells (Figure 5G). This result suggests that, in the absence of wounding, myeloid cells contribute to tumour vascularization and invasion in an inflammation-independent physiological manner.

### Physiological neutrophil migration conditions the collagen matrix for tumour cell invasion

**Figure 6:**
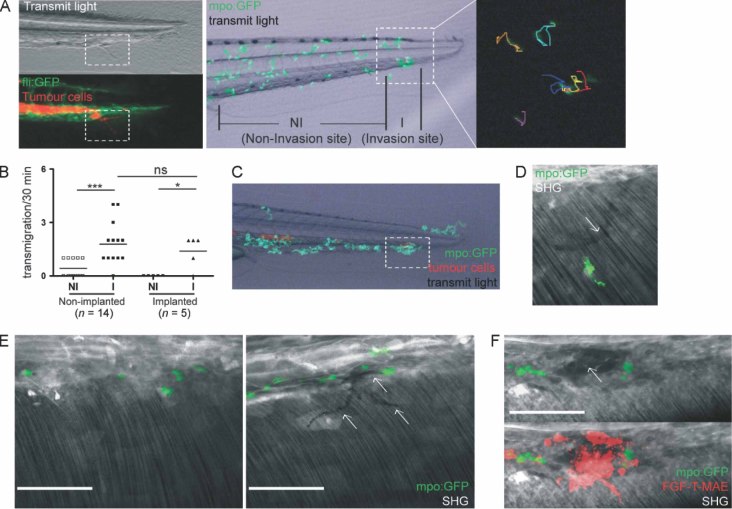
Physiological migration of neutrophils between the caudal haematopoietic tissue and the tail fin at the tumour invasion site conditions the collagen matrix for tumour cell invasion. (A) Migration tracks of GFP^+^ neutrophils in the tail of a Tg(mpx:GFP)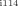 embryo (30 min time-lapse with 1 min intervals). GFP^+^ neutrophils only transmigrated between the caudal haematopoietic tissue (CHT) and the tail fin (TF) at the site where tumour cells invaded into the tail fin. The CHT–TF transmigration area is indicated in the left panel, which shows invasion of the tail fin by a dsRed-labelled FGF-T-MAE cell in a 3dpf Tg(fli:GFP) embryo (top, bright-field; bottom, fluorescence image). Manual tracking of 30 min migration patterns (right panel) of GFP^+^ neutrophils in the highlighted region of the tail of a 3dpf Tg(mpx:GFP)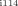 embryo (middle panel) showed that the transmigrating neutrophils (red, yellow and blue tracks) migrated as randomly as neighbouring neutrophils in the tail (orange and light blue tracks) or already inside the fin tissue (purple track). (B) Quantification of neutrophil transmigration into the tail fin. Time-lapse recordings were made of 3dpf Tg(mpx:GFP)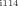 embryos with (*n* = 5) or without (*n* = 14) implanted FGF-T-MAE tumour cells, and the number of times that GFP^+^ neutrophils crossed the CHT–TF boundary was counted in two areas of the tail, the tumour invasion site (I) and the non-invasion site (NI), as indicated in (A). The CHT–TF transmigration of neutrophils selectively occurred at the same site as tumour invasion. This spatial preference was not influenced by implantation. Data are representative images of > three independent, reproducible experiments (> 30 embryos examined in total). (C) Recording (2 h) of neutrophil (green) migration in Tg(mpx:GFP)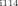 embryos after implantation of FGF-T-MAE tumour cells (red). The selective neutrophil CHT–TF transmigration site, overlapping with the tumour invasion site, is outlined. (D–F) Second-harmonic generation (SHG) analysis of the fibrillar collagen matrix around the CHT–TF transmigration site. Images show an overlay of SHG signal (grey) with fluorescence of GFP^+^ neutrophils [Tg(mpx:GFP)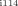] and red CM-DiI-labelled FGF-T-MAE tumour cells. (D) Example of a track (indicated by arrow) of deformed collagen matrix in the tail fin created by a migrating neutrophil. (E) Comparison of a region in the tail fin with no recent neutrophil migration events (left) and a region showing SHG-negative tracks (indicated with arrows) generated by migrating neutrophils (right). (F) Invasion of tumour cells in an SHG-negative site in the tail fin: (top) overlap of SHG and green fluorescence signal of neutrophils; (bottom) red fluorescence signal of tumour cells. The local loss of SHG signal is indicated by the arrow in the top image. Images in (A, C) were acquired using a Leica MZ16FA stereo microscope; those in (D, E) were acquired using a Zeiss 710 NLO upright two-photon confocal microscope with a × 20 water objective. Scale bars = 50 µm

To further investigate the involvement of neutrophils in tumour cell invasion, the physiological migration of neutrophils in 3dpf embryos was analysed using the Tg(mpx:GFP)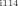 neutrophil-specific zebrafish reporter line 26. Time-lapse and trajectories analysis showed that neutrophils randomly transmigrated between the caudal haematopoietic tissue and the tail fin (termed ‘CHT–TF transmigration’), as a part of their physiological unrestricted random migration. This non-directional motion of neutrophils occurred selectively at the posterior end of the CHT, where the perivascular tumour cells invaded into the tail fin (Figure 6A, B). The spatial preference for this location of CHT–TF transmigration was not altered by tumour cell implantation (Figure 6B, C) or by wound-induced inflammation (unpublished data and 43, 44), suggesting that it is the normal physiological property of neutrophil migration in zebrafish embryos.

To further decipher the impact of neutrophil CHT–TF transmigration on the spatially co-localized tumour cell invasion process, two-photon excited confocal microscopy and SHG was performed to visualize the extracellular fibrillar collagen matrix upon neutrophil migration 38. Migrating neutrophils created transient tracks of deformed collagen matrix, as detected by the loss of SHG signal (Figure 6D), which was previously reported to be sufficient to support tumour cell invasion in mice models 45. As a consequence of the localized neutrophil CHT–TF transmigration, the deformation of the collagen matrix was largely enhanced at the posterior end of the CHT, where the perivascular tumour cells invaded into the tail fin (Figure 6E). Furthermore, we observed that the tumour cells invaded into the tail fin at the exact site of local collagen deformation (Figure 6F). Thus, the observed tumour cell invasion was promoted by physiological neutrophil migration (the CHT–TF transmigration), which conditions the extracellular collagen matrix.

**Figure 7:**
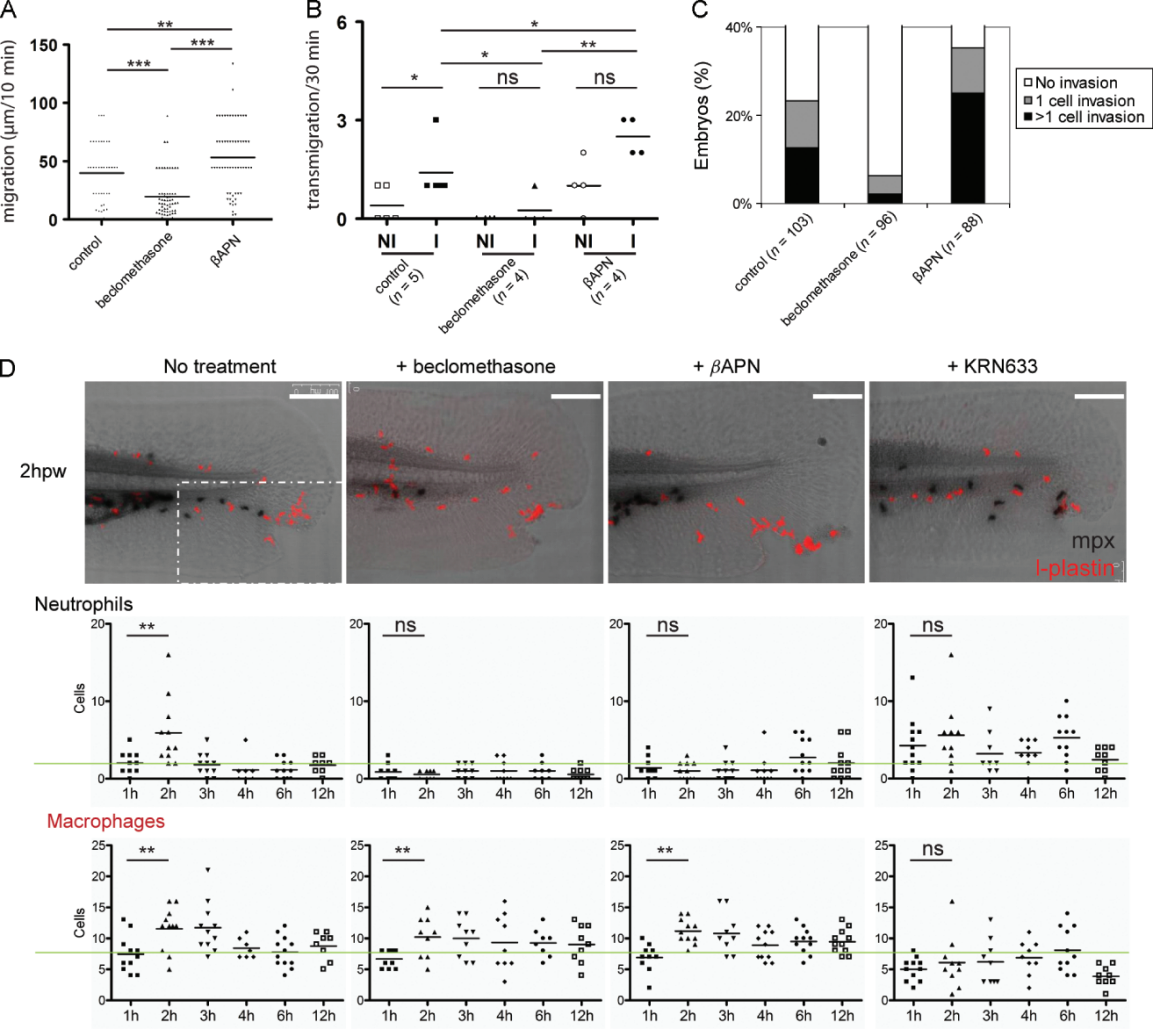
Tumour cell invasion is controlled by non-pathological neutrophil migration. (A) The random non-pathological migration of neutrophils was suppressed by beclomethasone (25 µm) and promoted by βAPN (500 µm). Migration tracks (• • µ m) of neutrophils in 3dpf Tg(mpx:GFP)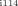 embryos were recorded over 10 min intervals (*n* ≥ 5 embryos/treatment group; *n* > •• total neutrophil tracks/treatment group). (B) The CHT–TF transmigration of neutrophils was suppressed by beclomethasone and promoted by βAPN. Transmigration events in non-invasion (NI) and invasion (I) areas of the tail fin were quantified as in Figure 6B. (C) Tumour cell invasion at the posterior end of the CHT was significantly suppressed by beclomethasone (Wilcoxon test, *p* < 0.01) and promoted by βAPN (*p* < 0.01). Embryos were scored for tumour cell invasion at 1dpi. Data are representive of > five independent experiments (each *n* > 60). (D) Wounding-induced myeloid cell chemotaxis was affected by different pharmacological treatments. 3dpf embryos were wounded in the tail fin, as indicated by arrows in representative images of embryos from different treatment groups (no treatment; 25 µm beclomethasone; 500 µm βAPN; 1 µm KRN633) at 2 h post-wounding (hpw). Embryos were fixed at different time points over a course of 12hpw and stained for neutrophils (Mpx^+^, histochemical staining) and macrophages (Mpx^−^/L-plastin^+^). Quantification of neutrophil and macrophage numbers was performed on *n* ≥ 8 embryos/time point for each treatment group. Images were acquired using a Leica TCS SPE confocal microscope with a × 20 dry objective. The macrophages/neutrophils in the region outlined were quantified to measure the myeloid cell chemotaxis towards the wound. Scale bars = 100 µm

Pharmacological approaches were taken to confirm the functional involvement of neutrophil migration in tumour cell invasion. A prototype glucocorticoid receptor agonist, beclomethasone, previously shown to inhibit neutrophil migration 46, did not cause alteration of the fibrillar collagen matrix in zebrafish embryos (data not shown). We detected an inhibition of the normal physiological neutrophil migration and of neutrophil chemotaxis directed by wounding-induced inflammation 39 at 4 h after administration of 25 µm beclomethasone (Figure 7A, B, D). Tumour cell invasion and micrometastasis was also reduced in embryos incubated in 25 µm beclomethasone 4 h before implantation (Figure 7C). In addition, we found that the lysyl oxidase inhibitor β-aminoproprionitrile (βAPN) largely reduced fibrillar collagen (unpublished data) and enhanced the CHT–TF transmigration of neutrophils, leading to a significant increase of tumour cell invasion and subsequent formation of micrometastases (Figure 7A–C). Notably, βAPN inhibited neutrophil chemotaxis induced by inflammation (Figure 7D), indicating that the increase of tumour cell invasion in βAPN-treated embryos was correlated with enhanced non-pathological neutrophil migration, but not with inflammation. Neither beclomethasone nor βAPN had any significant effect on macrophage chemotaxis (Figure 7D) and tumour vascularization (Figure 9I). Taken together, these results reveal that only non-pathological neutrophil migration and the spatially restricted CHT–TF transmigration modify the extracellular matrix and have a major impact on tumour cell invasion and micrometastasis in this model.

### VEGFR inhibitors suppress tumour vascularization, but promote tumour cell invasion by enhancing neutrophil migration

**Figure 8:**
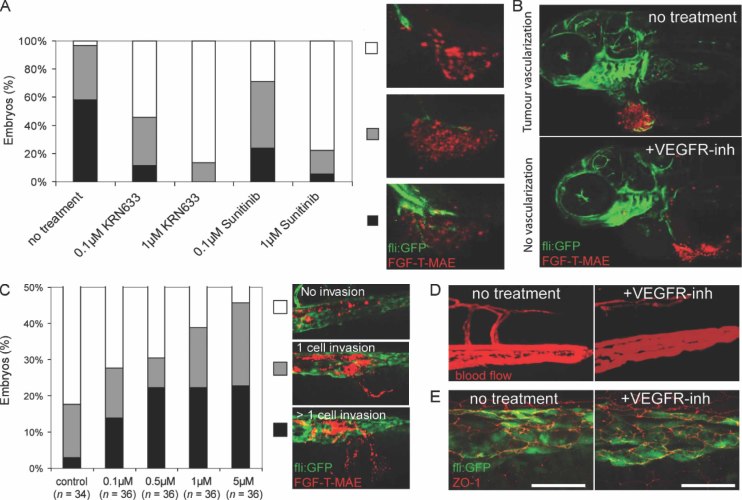
VEGFR inhibitors suppress tumour vascularization but promote tumour cell invasion. (A) Tumour vascularization was significantly suppressed by VEGFR inhibitors KRN633 or Sunitinib at both concentrations (Wilcoxon test, *p* < 0.01). The results were scored at 1dpi as the percentage of Tg(fli:GFP) embryos showing no (white), minor (grey) and extensive (black) neovasculature formation towards the FGF-T-MAE tumour cell mass. Representative images of each class are shown. Data are representative of > three independent experiments (each *n* > 30). (B) Expansion of the localized tumour mass was inhibited by 0.1 µm VEGFR inhibitor KRN633 or Sunitinib. Images are representative of > five independent experiments (each *n* > 30). (C) Tumour cell invasion at the posterior end of the CHT was significantly enhanced by VEGFR inhibitor KRN633 (Wilcoxon test, *p* < 0.01 for 0.5 µm). The results were scored at 1dpi, as the percentage of embryos showing phenotypes of no invasion (white), 1 cell invasion (grey) and > 1 cell invasion (black). Representative images of each class are shown. Data are representative of ≥ 10 independent experiments (each *n* > 30). (D) Microangiography using tetramethylrhodamine dextran. No leakage was detected at 24 h after administration of 0.1 µm VEGFR inhibitor KRN633 or Sunitinib. Data are representive of three independent experiments (each *n* > 3). (E) Immunohistochemistry against the tight junction protein ZO-1. Normal junctions were detected between endothelial cells at the posterior end of the CHT at 24 h after administration of 0.1 µm VEGFR inhibitor KRN633 or Sunitinib. Data are representative of three independent experiments (each *n* > 5). Scale bars = 25 µm. Images were acquired using a Leica TCS SPE confocal microscope with a × 20 dry objective (A–D) or a × 63 water objective (E)

Tumour vascularization is an important process in tumour progression. In our model, the localized tumour growth was determined by successful vascularization (Figure 2). Administration of the VEGFR inhibitors KRN633 or Sunitinib significantly inhibited tumour-induced neovasculature formation, in a dose-dependent manner (Figure 8A). Such inhibition of tumour vascularization resulted in significant suppression of localized tumour growth (Figure 8B). However, we noticed that tumour cell invasion at the site of CHT–TF transmigration was strongly promoted in embryos treated with the VEGFR inhibitors (Figure 8C). This observation supports the recent findings that anti-angiogenic therapies based on inhibition of VEGF signalling promote tumour cell invasion and metastasis in mouse models and in the clinic 7, 8, 16, 47. It is unlikely that such enhanced aggressiveness was caused by an effect on blood vessel integrity in our model, as no blood vessel leakage in these embryos was detected by microangiography using tetramethylrhodamine dextran (Figure 8D). Furthermore, immunohistochemistry with an antibody against the tight junction protein ZO-1 showed that endothelial cell–cell junctions remained normal after VEGFR inhibition (Figure 8E).

**Figure 9:**
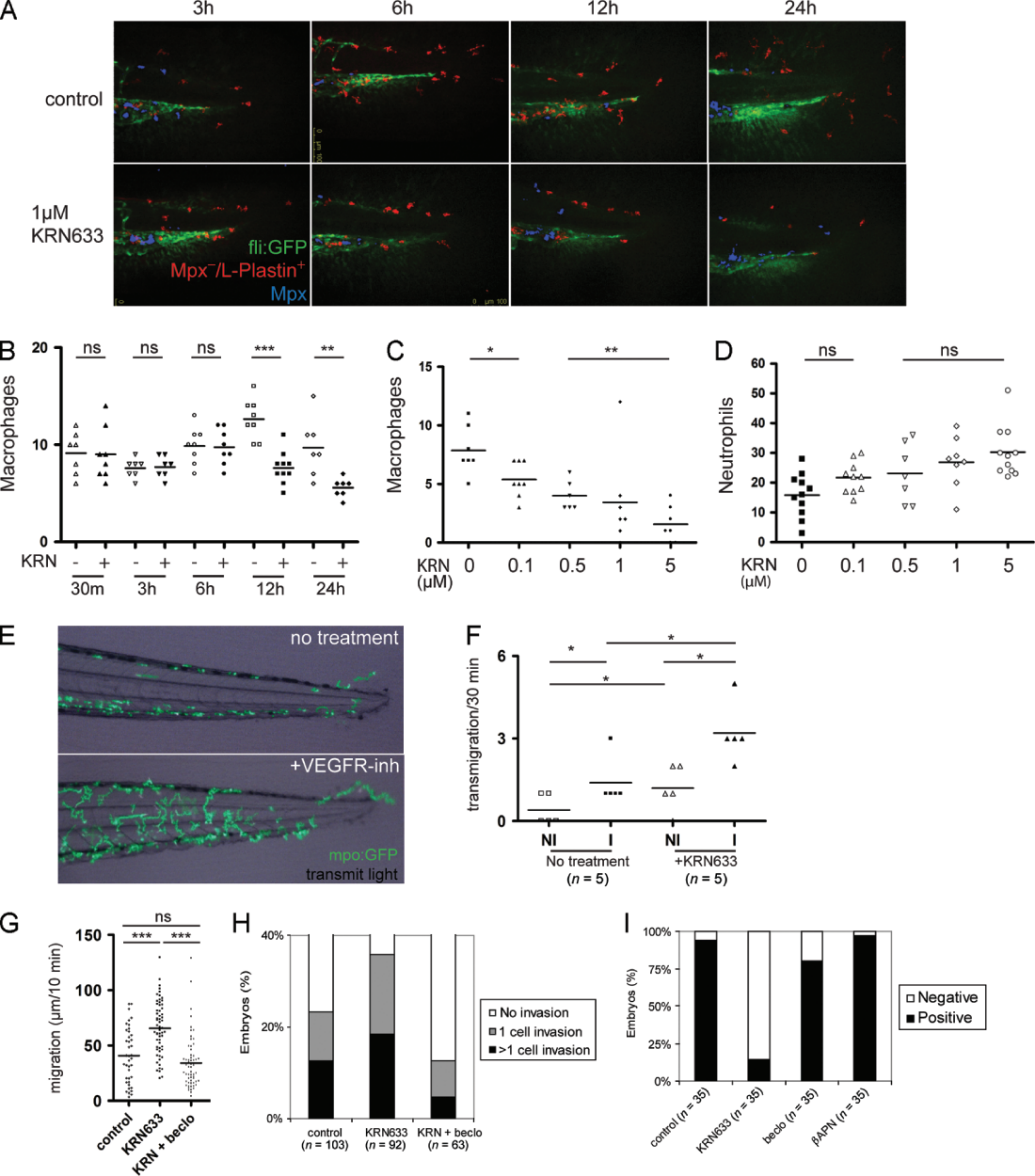
VEGFR inhibitors promoted tumour cell invasion via enhancement of physiological neutrophil migration. (A–C) Suppression of macrophages by VEGFR inhibition. 2dpf Tg(fli:GFP) embryos were untreated or treated with 1 µm KRN633 and subsequently stained for neutrophils (Mpx^+^, histochemical staining, shown in blue) and macrophages (Mpx^−^/L-plastin^+^, shown in red). Myeloid cells in the tail of representative embryos were quantified in a 0.5 mm region from the tip of the tail fin. Representative embryos were imaged at the last 0.5 mm from the tip of the tail fin and counted for myeloid cells. Notably, the elongated morphology of macrophages was altered to a less migratory phenotype (rounded morphology) after 6 h of treatment (A) and the number of macrophages in this region was irreversibly reduced after 12 h (B). 24 h administration of KRN633 caused reduction of macrophages in a dose-dependent manner (C). Images were acquired using a Leica TCS SPE confocal microscope with a × 20 dry objective. Data are representative of three independent experiments. (D) KRN633 did not reduce neutrophil numbers after 24 h administration. (E) Administration of 0.1 µm VEGFR inhibitor KRN633 or Sunitinib resulted in elongated neutrophil [Tg(mpx:GFP)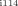] migration tracks over a 30 min time frame. The time-lapse series were acquired using a Leica MZ16FA stereo microscope. (F) The CHT–TF transmigration of neutrophils was promoted by 0.1 µm KRN633 (*n* = 5). Transmigration events in non-invasion (NI) and invasion (I) areas of the tail fin were quantified as in Figure 6B. (G) The random non-pathological migration of neutrophils was promoted by 0.1 µm KRN633. KRN633-promoted migration was suppressed by co-administration of 25 µm beclomethasone. Migration tracks (µ m) of neutrophils in 3dpf Tg(mpx:GFP)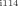 embryos were recorded over 10 min intervals. Data are representative of two independent experiments (*n* > 5 embryos/treatment group; *n* > 20 total neutrophil tracks/embryo). (H) Tumour cell invasion promoted by 0.1 µm KRN633 was significantly suppressed by 25 µm beclomethasone (Wilcoxon test, *p* < 0.01). Tumour cell invasion was quantified as in Figure 8C. Data are representative for three independent experiments (each *n* > 60). (I) Tumour vascularization was significantly suppressed by 0.1 µm KRN633 (Wilcoxon test, *p* < 0.01) but not by 25 µm beclomethasone or 500 µm βAPN. Embryos were scored for vasculogenesis at 1dpi, as in Figure 5C. Data are representative of > three independent experiments (each *n* > 30)

We then investigated the contribution of myeloid cells to the tumour cell invasion and micrometastasis promoted by VEGFR inhibition. We found that VEGFR inhibition led to a reduction of macrophage numbers, altered the typical elongated macrophage shape into a rounded morphology and suppressed the migratory activity of macrophages (Figures 7D, 9A–C). In contrast, the number of neutrophils was not reduced by VEGFR inhibition (Figure 9D). Furthermore, the random migration and CHT–TF transmigration of neutrophils were enhanced upon VEGFR inhibition, as shown in live recordings (Figure 9E–G). These results suggest that VEGFR inhibition enhanced neutrophil migration, which in turn promoted tumour cell invasion. To test this hypothesis, beclomethasone was administered to achieve a pharmacological restoration of neutrophil migration upon VEGFR inhibition (Figure 9G). Subsequently, the tumour cell invasion promoted by VEGFR inhibition was also restored to the normal level (Figure 9H). These results confirm that the VEGFR inhibitors promoted tumour cell invasion and micrometastasis by the enhancement of neutrophil migration.

## Discussion

The recent discoveries in mice models and clinical studies showed that anti-angiogenic therapies by targeting the VEGF signalling inhibited primary tumour growth but promoted tumour invasion and metastasis. Unfortunately, the mechanism of this very relevant clinical problem is still elusive. Answering this question requires live imaging of tumour-microenvironment interactions at the cellular level—a process severely limited in current animal models.

Using a novel zebrafish xenograft model that allows simultaneous single-cell resolution monitoring of multi-step tumourigenesis *in vivo*, including tumour vascularization, localized tumour growth, tumour invasion and micrometastasis formation, we discovered a new mechanism of the metastatic niche formation. In addition to the published roles of macrophages in this process 14, we found that physiological migration of neutrophils controlled tumour invasion by conditioning the collagen matrix to facilitate the metastatic niche. Importantly, we show that VEGFR inhibitors suppress localized tumour growth but, in contrast, promote tumour invasion and micrometastasis formation by enhancing neutrophil migration.

Recently, the zebrafish myeloid cell population has been studied in detail and similarities with the human myeloid cell functions and embryonic differentiation have been demonstrated 48, 49. Further evidence that the zebrafish holds much potential for translational studies in cancer has been published 50, 51. Recently it has been reported that expression of WHIM truncation mutations of CXCR4 in zebrafish neutrophils induces neutrophil retention in the caudal haematopoietic tissue, which represents a mechanism of human immunological WHIM disorder. WHIM zebrafish are neutropenic, and WHIM neutrophils show impaired recruitment to tissue inflammation sites, recapitulating the human disease. These findings support the potential of zebrafish to model primary immune disorders and underscore the translational relevance of zebrafish neutrophils transmigration in understanding disease pathogenesis 52, 53.

The involvement of myeloid cells in critical tumourigenesis, steps such as vascularization and invasion, was revealed by both genetic and pharmacological approaches. We demonstrated that myeloid cells, particularly macrophages, are essential for tumour vascularization. Importantly, we also obtained evidence that the micrometastasis site was determined by the physiological migration of neutrophils. Our results show that yet undefined environmental cues favour neutrophil transmigration at the posterior end of the caudal haematopoietic tissue, which remodels the fibrillar collagen matrix and in turn conditions the premetastatic niche for tumour cells to invade into the tail fin. These findings support the ‘seed and soil’ hypothesis, that tumour metastasis is controlled by both the tumourigenic property of disseminated cells and their microenvironment 54.

It is known that tumour-infiltrating immune cells can have both tumour-promoting and tumour-antagonizing roles 1. In our model, the myeloid cells promote tumour progression under the physiological wound-free condition (Figure 5C, E), whereas immune-mediated destruction of tumour cells was hyper-activated by wound-induced inflammation (Figure 5F, G). These results indicate that environmental factors have an impact on the balance between the conflicting immune cell responses to tumour cells. Interestingly, we found that the lysyl oxidase inhibitor βAPN accelerates physiological neutrophil migration but decelerates pathological neutrophil chemotaxis, suggesting that different molecular mechanisms are involved in different modes of myeloid cell migration.

Our data support the recent notion that inhibition of VEGF signalling may have opposite effects on localized tumour growth and on metastasis. It has been suggested that hypoxia-resistant cells may escape from a starving primary tumour treated with VEGFR inhibitors and then result in elevated malignancy 2, 8, 12, 47. In our model, micrometastasis originates from tumour cells disseminated haematogenously directly after implantation, and therefore hypoxia is unlikely to be the mediator of enhanced tumour invasiveness upon VEGFR inhibition.

Our data provide evidence that the physiological migration of neutrophils is elevated upon inhibition of VEGF signalling, which in turn promotes tumour invasion. This is the first demonstration, to our knowledge, of the *in vivo* cooperativeness between VEGF signalling and neutrophils in metastasis. In addition, we observed that macrophages were suppressed by the VEGFR inhibitors, which may indicate an indirect involvement of macrophages in this process. The remaining question is, what are the targets of the VEGFR inhibitors in the neutrophils that cause them to enhance their migration? The VEGFR inhibitors used in this study are sufficiently different in structure to ensure that the observations were not due to an off-target effect. However, we can not be certain that observed phenotypes are restricted to targeting of VEGF receptors, since sunitinib works also on PDGFR family members and affects pericyte coverage of endothelium, promoting extravasation of tumour cells and the formation of micrometastasis 55. It is unlikely that the enhanced aggressiveness was caused by an effect on blood vessel integrity in our model, as no blood vessel leakage in these embryos was detected by microangiography using tetramethylrhodamine dextran. Unfortunately, pericytes have not yet been described in zebrafish and thus we were unable to look at them directly.

Our findings implicate myeloid cells as critical mediators of tumour invasiveness elicited by VEGFR inhibitors, independent of inflammation and the adaptive immune system. It provides a new mechanism underlying the recent clinical observations of anti-VEGF therapies promoting tumour invasiveness. We propose that dual targeting of angiogenesis and myeloid cells can be beneficial in future treatments. Of course, it will only be relevant for medical studies if the neutrophil transmigration is not restricted to this model. Currently the role of macrophages in intra- and extravasation of cancer cells is well established 14. The role of neutrophils is less studied; however, tumour-associated neutrophils (TANs) were recently described and an increased level of neutrophils in cancer patients was shown to correlate with poor prognosis 56. For example, a quantification of TANs in patients with renal cell carcinoma revealed that the presence of neutrophils correlated with increased mortality 56. In addition, increased levels of TANs in the bronchioalveolar space of patients with bronchioalveolar carcinoma were significantly associated with poor outcomes 57. In order to dissect the function of myeloid cells in human cancers, live imaging of tumour–microenvironment interactions at the cellular level is required, a process severely limited in current mammalian models. This limitation strongly supports the relevance of the zebrafish model to unravel the molecular mechanisms of how tumour-associated neutrophils mediate metastasis in zebrafish and mammalian models. Furthermore, our zebrafish xenotranplantation system provides a new, inexpensive animal model for rapid preclinical screening of new potential therapies, for example directed at the VEGF signalling pathway, at various stages of tumour progression and metastasis spread in real time in an *in vivo* model. In conclusion, we exploited this model here to demonstrate that neutrophil migration affects metastasis and that this is enhanced by VEGFR inhibitors.
